# BAX and SMAC regulate bistable properties of the apoptotic caspase system

**DOI:** 10.1038/s41598-021-82215-2

**Published:** 2021-02-08

**Authors:** Stephanie McKenna, Lucía García-Gutiérrez, David Matallanas, Dirk Fey

**Affiliations:** 1 Systems Biology Ireland, University College Dublin, Belfield, Dublin 4, Ireland; 2 School of Medicine, University College Dublin, Belfield, Dublin 4, Ireland

**Keywords:** Computational biology and bioinformatics, Molecular biology, Systems biology

## Abstract

The initiation of apoptosis is a core mechanism in cellular biology by which organisms control the removal of damaged or unnecessary cells. The irreversible activation of caspases is essential for apoptosis, and mathematical models have demonstrated that the process is tightly regulated by positive feedback and a bistable switch. BAX and SMAC are often dysregulated in diseases such as cancer or neurodegeneration and are two key regulators that interact with the caspase system generating the apoptotic switch. Here we present a mathematical model of how BAX and SMAC control the apoptotic switch. Formulated as a system of ordinary differential equations, the model summarises experimental and computational evidence from the literature and incorporates the biochemical mechanisms of how BAX and SMAC interact with the components of the caspase system. Using simulations and bifurcation analysis, we find that both BAX and SMAC regulate the time-delay and activation threshold of the apoptotic switch. Interestingly, the model predicted that BAX (not SMAC) controls the amplitude of the apoptotic switch. Cell culture experiments using siRNA mediated BAX and SMAC knockdowns validated this model prediction. We further validated the model using data of the NCI-60 cell line panel using BAX protein expression as a cell-line specific parameter and show that model simulations correlated with the cellular response to DNA damaging drugs and established a defined threshold for caspase activation that could distinguish between sensitive and resistant melanoma cells. In summary, we present an experimentally validated dynamic model that summarises our current knowledge of how BAX and SMAC regulate the bistable properties of irreversible caspase activation during apoptosis.

## Introduction

Correct regulation of cellular homeostasis is fundamental in multicellular organisms. Thus, this process is tightly regulated by complex molecular networks which control cell fate, determining when a cell should proliferate, differentiate or die through programmed cell death. Apoptosis is an essential form of programmed cell death which mediates the removal of damaged or unnecessary cells. Tight regulation of apoptosis is vital to the maintenance of normal cell function. Apoptosis can proceed via the extrinsic pathway, induced by binding of an extracellular ligand to its death receptor, or the intrinsic pathway, activated by a diverse array of cytotoxic stimuli which converge on the outer mitochondrial membrane^1,2^. Subsequent release of pro-apoptotic proteins from the mitochondria is regulated by pro-apoptotic and anti-apoptotic BCL2 proteins^2^. Both apoptotic pathways are mediated by the cleaving proteins, caspases, which are produced as inactive zymogens^3^. Initiator caspases (2, 8, 10, 9) are activated by pro-apoptotic proteins and subsequently cleave and activate executioner caspases (3, 6, 7) which act directly on cell dismantling substrates^2,3^. Apoptosis is triggered when the caspase cascade is irreversibly activated, and several proteins regulate the correct switching on of this machinery to ensure avoidance of incomplete cell death. Positive and negative regulators of the caspases include the BCL2 family of proteins^4–7^, the inhibitor of apoptosis (IAP)^8–10^ family and several proteins such as APAF^11^, Cytochrome C (CytC)^5,7,12^ and SMAC (Second mitochondria-derived activator of caspases), also called Direct IAP-binding Protein with low pI (DIABLO^5,12–15^, hereto SMAC). Importantly, dysregulation of apoptosis is common in disease, and is a hallmark of cancer and neurodegenerative diseases^16,17^.

Given its central role in life, the mechanisms that regulate apoptotic networks are the focus of intensive research. This work has shown that there are several control points which determine the activation of apoptosis upon pro-apoptotic signals^5^. Given the complex dynamics regulating these mechanisms, several groups have applied systems biology approaches to study the mechanistic regulation of these apoptotic networks^18–24^. It has been shown that the relative stoichiometry of the different proteins, caspase cleavage and protein degradation rates determine the commitment of the apoptotic machinery to cell death. In the case of the intrinsic pathway, it has been experimentally and computationally demonstrated that caspase dependent apoptosis is a highly controlled process which exhibits an irreversible, bistable switch^18–20,25^. Mathematically, bistability is the coexistence of two stable steady states, allowing for rapid, often irreversible transition between these steady states. Once a stimulus threshold is exceeded, the system can switch from a low level of activation (the ‘off’ stable steady state) to a high level of activation (the ‘on’ stable steady state) in an all-or-nothing fashion^20^. Phenomenologically, a bistable switch exhibits two characteristic properties. On the level of the dose–response: the emergence of the hysteresis effect which implies that for the system to switch “on” from one steady state to the other, the input stimulus must exceed a certain threshold. The system will reside in this new steady state, even if the stimulus changes. In order to switch “off” to the preceding steady state, a different, lower stimulus threshold must be reached. The hysteresis effect indicates that the system maintains different on and off thresholds. On the level of the time-course, a characteristic time-delay precedes the rapid switch-like transition to the other stable steady-state. Upon application of the input stimulus, the system remains relatively unchanged in the “off” state for some amount of time before rapidly activating in a snap-like fashion. This time-delay of activation can be increased or decreased by alteration of the stimulus concentration or modification of model components.

The primary mechanism generating bistability in the intrinsic pathway is positive feedback^20^. This positive feedback is realised by an explicit and implicit feedback loop. Explicitly, upon cleavage and activation of caspase 3 by caspase 9, caspase 3 can in turn cleave and activate caspase 9^20^. Considering that XIAP is the IAP protein which maintains the important caspase binding residues and other IAPs such as cIAP do not have caspase inhibiting activity^26^, the model focused on XIAP. In the implicitly hidden feedback loop, XIAP can bind and inhibit all caspases within the cascade. Caspase 3 relieves inhibition of its upstream activator caspase 9 through XIAP binding, thereby creating a second, implicit positive feedback loop to contribute to the bistability of the system^20^. Previous work indicates that the explicit and implicit feedback mechanisms could be an important feature in determining how the apoptotic system achieves ultra-sensitivity, bistability and irreversibility. In order to get a better understanding of the properties of the bistable switch in the intrinsic apoptotic pathway and understand how its deregulation may be related to disease it is necessary to develop models that include other regulatory proteins of the network. Models developed including BAX and SMAC have shown that these proteins are key in the regulation of apoptotic commitment. BAX is the gateway to mitochondrial activation of intrinsic apoptosis and has been shown to form pores in the mitochondrial membrane resulting in the release of pro-apoptotic proteins^27–29^. SMAC is one of the proteins released from the mitochondria upon activation of the intrinsic apoptotic pathway and is an inhibitor of IAPs^30^. Correct function of both BAX and SMAC is central to the regulation of apoptosis. Importantly, deregulation of these pro-apoptotic proteins has been described in tumours^6,27,29,31–40^. But how these patient-specific differences of BAX and SMAC expression affect the properties of the bistable switch, such as the amplitude of caspase activation and the sensitivity of tumour cells to DNA damaging drugs, is not well understood. Understanding these quantitative effects requires the development of a mathematical model because the bistable caspase system behaves highly non-linear and because BAX and SMAC enter the network topology at different nodes.

Here, we wanted to understand how BAX and SMAC control the emergent properties of the bistable switch and test whether a mathematical model of this switch could predict the sensitivity of melanoma cell lines to DNA damaging drugs. Thus, we have built a mathematical model of BAX and SMAC interactions with the caspase system which mirrors experimental observations from our own experiments and the literature. Here we show that once the caspase 3 activation threshold is reached, caspase 3 switches to the high activity ‘on’ state in an irreversible, all-or-nothing manner, which is in line with previously published models^18–20,22,41^. We demonstrate that upon increasing the input, BAX expression level or SMAC release rate were shown to reduce the time lag in caspase 3 activation. Our bifurcation analyses revealed that an increase in BAX expression resulted in a reduction in the caspase 3 activation threshold. An increase in SMAC release rate also exhibited a reduction in caspase 3 activation threshold, though to a lesser extent. Simulations confirmed by validation experiments show that the caspase system might be differentially sensitive to BAX and SMAC mimetics. Finally, we further validated the model on data of the NCI-60 cell line panel using BAX protein expression as a cell-line specific parameter. The model simulations correlated with the cellular response to DNA damaging drugs and established a threshold of caspase 3 activation that could classify melanoma cells into sensitive and resistant cells.

## Results

### Model construction

#### Dynamic modelling of BAX and SMAC regulated the intrinsic apoptotic pathway

**Figure 1:**
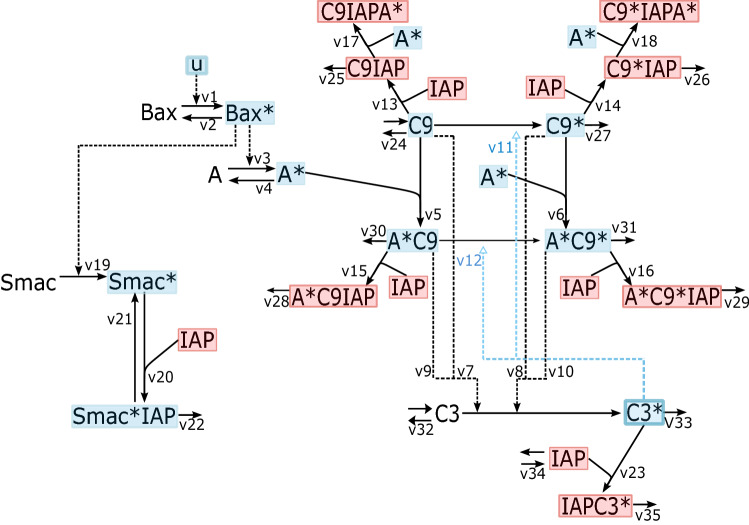
Reaction kinetic scheme of the intrinsic apoptosis model. A pro-apoptotic insult, u, activates BAX. Upon activation of BAX, heptamerisation of Apaf-1 (A) occurs to form the active apoptosome (A*), which by recruitment of caspase 9 forms complexes that can activate the intrinsic apoptosis cascade. A* can recruit and stimulate both pro-caspase 9 (C9) and cleaved caspase 9 (C9*). When active, caspase 9 causes cleavage and activation of pro-caspase 3 (C3). In turn, active caspase 3 (C3*) can activate caspase 9 through a positive feedback loop. Further, inhibitor of apoptosis protein (IAP) can bind to all forms of caspase 9 and caspase 3 and block their activity. Active SMAC* is released by Bax* and can bind the free form of IAP leading to the degradation of the SMAC*IAP complex. * denotes the active form of the protein.

Our main aim was to test whether a mathematical model of the apoptotic switch as regulated by BAX and SMAC could predict the sensitivity of cancer cells, particularly melanoma cells, to DNA damaging drugs. To construct the model, we extended the model of caspase activation by Legewie et al.^20^, which is based on ordinary differential equations, with information from the literature to include (i) BAX mediated initiation of the caspase cascade and (ii) SMAC-mediated sequestration of XIAP^42–44^. The scope of the model was limited to BAX and SMAC because of their clinical significance and importance in regulating the caspase system. Other potentially important regulators such as the BAX analogue BAK and BCL2 proteins were not included because their protein expression levels did not correlate with the response to DNA damaging drugs in the NCI60 cell line panel (Supplementary Figs. 6, 7). The reaction kinetic scheme of the model is outlined in Fig. 1. The expansion of the model to include BAX and SMAC is detailed below.

In the Legewie model, caspase activation is initiated by apoptosome (denoted A* in the model, Fig. 1) formation through heptamerisation of APAF-1 and binding to procaspase 9 resulting in procaspase 9 autoproteolysis and activation. Active procaspase 9 (C9) then causes caspase 9 (C9*) cleavage and activation. In accordance with the well calibrated Legewie model and the references therein^20^ it has been assumed that A* can recruit and stimulate both C9 and C9*; C9* can activate procaspase 3 (C3) which can in turn cleave and activate caspase 3 (C3*) and this C3 cleavage rate is much higher when C9* is bound to A*. C3* can activate caspase 9 through a positive feedback loop. XIAP (X), which maintains an E3 ligase domain, can reversibly bind and inhibit all forms of caspase 9 and caspase 3^15^ to prevent the activation of cell death.

An important limitation of this model is that it does not consider evidence that shows that Cytochrome C (CytC ) release from the mitochondria is dependent on BAX pore formation^7^ and further that CytC release itself is insufficient to completely prevent XIAP’s inhibitory effect upon caspases which requires the participation of SMAC^33^. Thus, the model was expanded to include apoptotic regulators, BAX and SMAC. BAX becomes activated (BAX*) by a pro-apoptotic insult (u). To keep the model simple the depolarisation of the mitochondrial membrane by BAX, the release of CytC from the mitochondria and the subsequent activation of APAF-1 causing formation of the apoptosome (A*), was lumped into a single reaction in which A* formation is dependent on active BAX. Concomitantly, SMAC is released from the mitochondria in a BAX* dependant manner to bind and form an inhibitory complex with XIAP, which can be degraded^12,14^. More details about the modelling of BAX and SMAC are provided in two dedicated sections below.

A full account of all reactions, rate laws, parameters and equations, including initial conditions is given in Tables 1 and 2. This ODE model provides a mathematical representation to describe exactly how a pro-apoptotic insult activates the intrinsic apoptosis pathway and how these species change over time. The model can be used to simulate how cell-line and patient-specific differences of gene and protein expression may alter the system dynamics of caspase activation—predictions which may be experimentally validated.

**Table 1:** Table of model reactions and reaction rates.

Number	Reaction	Reaction rate	k+ (1/s)	k- (1//s)	Source
1	BAX + u →BAX* + u	k1 · u · BAX	1.00E−03	–	Estimated from Lindner et al, 2013
2	BAX*→BAX	k2 · BAX*	5.00E−03	–	Lindner et al, 2013
3	A + BAX* →A* + BAX*	k3 · A · BAX*	1.00E−03	–	Legewie et al, 2006
4	A*→A	k4 · A*	1.00E−03	–	Legewie et al, 2006
5	C9 + A*⟷A*C9	k5 · A · C9 - ki5 · AC9	2.00E−03	0.1	Legewie et al, 2006
6	C9* + A*⟷A*C9*	k6 · A · C9* - ki6 · AC9*	2.00E−03	0.1	Legewie et al, 2006
7	C3 + C9→C3* + C9	k7 · C3 · C9	5.00E−06	–	Legewie et al, 2006
8	C3 + C9*→C3* + C9*	k8 · C3 · C9*	5.00E−05	–	Legewie et al, 2006
9	C3 + A*C9 →C3* + A*C9	k9 · C3 · A*C9	3.50E−04	–	Legewie et al, 2006
10	C3 + A*C9* →C3* + A*C9*	k10 · C3 · A*C9*	3.50E−04	–	Legewie et al, 2006
11	C3* + C9 →C3* + C9*	k11 · C3* · C9	2.00E−04	–	Legewie et al, 2006
12	C3* + A*C9→C3* + A*C9*	k12 · C3* · A*C9	2.00E−04	–	Legewie et al, 2006
13	C9 + X ⟷C9X	k13 · C9 · X - ki13 · C9X	1.00E−03	1.00E−03	Legewie et al, 2006
14	C9* + X ⟷C9*X	k14 · C9* · X - ki14 · C9*X	1.00E−03	1.00E−03	Legewie et al, 2006
15	A*C9 + X ⟷A*C9X	k15 · A*C9 · X - ki15 · A*C9X	1.00E−03	1.00E−03	Legewie et al, 2006
16	A*C9* + X ⟷A*C9*X	k16 · A*C9* · X - ki16 · A*C9*X	1.00E−03	1.00E−03	Legewie et al, 2006
17	C9X + A* ⟶A*C9X	k17 · C9X · A* - ki17 · A*C9X	2.00E−03	0.1	Legewie et al, 2006
18	C9*X + A*⟶A*C9*X	k18 · C9*X · A* - ki6 · A*C9*X	2.00E−03	0.1	Legewie et al, 2006
19	SMAC + BAX* ⟶SMAC	k19 · BAX*	1.00E−01	–	Estimated from Flanagan et al,2011
20	SMAC + X⟶SMACX	k20 · SMAC · X	1.00E−01	–	Flanagan et al, 2011
21	SMACX⟶SMAC + X	k20 · SMACX	1.00E−01	–	Flanagan et al, 2011
22	SMACX ⟶	k20 · SMAC X	1.00E−03	–	Estimated
23	C3* + X ⟷XC3*	k23 · C3* · X - ki23 · C3*X	3.00E−03	1.00E−03	Legewie et al, 2006
24	C9⟷	k24 - ki24 · C9	1.00E−03	0.02	Legewie et al, 2006
25	C9X⟷	k25 · C9X	1.00E−03	–	Legewie et al, 2006
26	C9*X→	k26 · C9*X	1.00E−03	–	Legewie et al, 2006
27	C9*→	k27 · C9*	1.00E−03	–	Legewie et al, 2006
28	A*C9X→	k28 · A*C9X	1.00E−03	–	Legewie et al, 2006
29	A*C9*X→	k29 · A*C9*X	1.00E−03	–	Legewie et al, 2006
30	A*C9→	k30 · A*C9	1.00E−03	–	Legewie et al, 2006
31	A*C9*→	k31 · A*C9*	1.00E−03	–	Legewie et al, 2006
32	C3⟷	k32 · C3 - ki32 · C3	1.00E−03	0.2	Legewie et al, 2006
33	C3*→	k33 · C3*	1.00E−03	–	Legewie et al, 2006
34	X⟷	k34 - ki34 X	1.00E−03	0.04	Legewie et al, 2006
35	C3*X→	k34 · C3*X	1.00E−03	–	Legewie et al, 2006
Conserved		BAX = BAXtot -BAX*	BAXtot = 0.5		
Moieties		A = Atot -A* -A*C9 -A*C9X -A*C9* -A*C9*X	Atot = 20

**Table 2:** Table of ordinary differential equations.

Left-hand sides	Right-hand sides	Initial concentrations (nM)
d[BAX*]/dt	v1 – v2	0
d[A*]/dt	v3 – v4 – v5 – v6 – v17 – v18 + v28 + v30 + v31 + v29	0
d[C9]/dt	– v5 – v11 – v13 + v24	20
d[C9X]/dt	v13 – v17 – v25	0
d[X]/dt	– v13 – v14 – v15 – v16 – v20 + v21 – v23 + v34	20
d[A*C9X]/dt	v15 + v17 – v28	0
d[A*C9]/dt	v5 – v15 – v30	0
d[C3]/dt	– v7 – v8 – v9 – v10 + v32	200
d[C3*]/dt	v7 + v8 + v9 + v10 – v23 – v33	0
d[C3*X]/dt	v23 – v35	0
d[C9*X]/dt	v14 – v18 – v26	0
d[C9*]/dt	v11 – v6 – v14 – v27	0
d[A*C9*]/dt	v6 + v12 – v16 – v31	0
d[A*C9*X]/dt	v16 + v18 – v29	0
d[SMAC]/dt	v19 – v20 + v21	0
d[SMACX]/dt	v20 – v21 – v22	0

In the following section we review experimental evidence for the BAX and SMAC mechanisms of crosstalk with the intrinsic apoptosis pathway in detail and show how they are implemented in the dynamic model. Finally, we explore the intricate kinetic behaviour and dynamics of BAX and SMAC-regulated caspase activation.

#### Modelling BAX

As already mentioned, as one of the most important proteins in the BCL2 pro-apoptotic family, BAX, mediates the activation of intrinsic apoptosis in response to DNA damage^45^. Upon pro-apoptotic insult, BAX is translocated to the mitochondrial membrane where it can homo-oligomerise to form the ‘apoptotic pore’ within the membrane^46^. This mitochondrial outer membrane permeabilization (MOMP) results in the release of CytC from the mitochondria, triggering activation of the caspase cascade^46^. Pro-apoptotic BCL2 proteins including BAX can be deactivated by binding of antiapoptotic BCL2 proteins, thereby blocking activation of the intrinsic apoptosis cascade^46^.

In our model BAX acts upstream of APAF-1 heptamerisation (Fig. 1). BAX activation and translocation are modelled in a single reaction (reaction v1, Fig. 1). Neglecting biological details such as the inhibitory binding and neutralisation of BCL-XL to BAX^7,31^, deactivation of BAX* is modelled as a simple first order reaction (v2, Fig. 1). Active BAX* subsequently mediates the formation of the apoptosome, which is modelled as a first order reaction, dependant on BAX* (v3, Fig. 1). A*subsequently triggers the activation of the caspase cascade.

#### Modelling SMAC

Following a pro-apoptotic insult and initiation of MOMP by the BAX mediated formation of pores in the mitochondrial membrane, SMAC is released from the mitochondria into the cytosol, where it binds and releases the inhibitory effect of XIAP on caspases^30^. When SMAC binds the second or third BIR domain of XIAP, its E3 ubiquitin ligase activity is stimulated, XIAP is ubiquitinated and inhibited^47^. Degradation of XIAP proteins following SMAC binding results in the loss of its inhibitory effect over procaspases 3 and 9^12^. In this way, SMAC relieves the inhibition procaspase 3 and mature caspase 3, thus promoting the induction of apoptosis^30^. Early evidence has shown that SMAC can exist as a dimer or monomer and that the dimer is essential for its pro-apoptotic role^30^. It was also shown that the N-terminal region of SMAC is necessary for the activation of caspase 3 and that a short peptide mimicking the N-terminus could mediate activation of apoptosis^30,33^, demonstrating the key role of this protein in the activation of the intrinsic apoptotic pathway. This has led to the development of a series of SMAC mimetics for the treatment of cancer that are being tested in the clinic^48^.

Based on this knowledge, and to include SMAC, we extended the core model by several processes. BAX* translocation to the mitochondria triggers MOMP-mediated SMAC release and dimerisation. To simplify the model, SMAC release and dimerisation was modelled as a single, first order reaction dependant on BAX* (v19, Fig. 1). Secondly, SMAC forms a complex with XIAP to eliminate XIAP inhibition of caspase activity (v20), relieving formation of inhibitory complexes with caspase 9 and caspase 3 (C9XIAP, C9*XIAP, A*C9XIAP, A*C9*XIAP, C3*XIAP). We have considered SMACXIAP complex formation to be a reversible reaction (v21) as has been previously modelled by Albeck et al^22^. Degradation of the SMACXIAP complex is represented by v22. We have made two assumptions in the modelling of SMAC dynamics. In line with previously published models^22^, we have assumed SMAC release is 10 times faster than activation of A* to capture the extra time required for the multi-step mechanism of A* formation that was simplified in the model. Secondly, and in accordance with previously published models, we have assumed that the parameter values for SMAC association with XIAP are similar to the caspase-XIAP interactions considering they bind at the same site^20,22^. Though, to ensure that SMAC has the ability to sequester XIAP away from both caspase 3 and caspase 9 as has been experimentally observed^44^, we have modelled SMAC-XIAP binding with slightly higher affinity.

#### The model simulates the experimentally observed timing and irreversibility of the apoptotic switch

**Figure 2:**
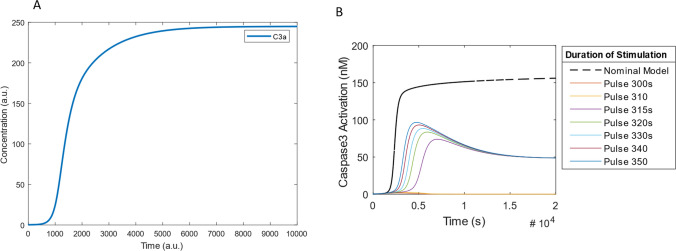
Investigating Model Behaviour. (**A**) Time course of activation of caspase 3 under constant stimulation. BAX was activated upon application of stimulus, *u*(0.5 nM). The time course demonstrates that the model simulation of caspase 3 activation is in accordance with experimental results—that caspase 3 is activated within 15 min following initiation^14^. (**B**) Time course of caspase 3 activation in response to transient pulse stimulation. Upon stimulation of BAX by *u*(0.5 nM) for 300 s, caspase 3 does not become activated. When a pulse stimulation of *u*(0.5 nM) for 315 s was applied, caspase 3 was activated to ~ 50% of nominal model caspase 3 activation. Once the stimulus is removed, caspase 3 remains activated demonstrating that once the stimulus threshold of 315 s is reached, caspase 3 activation is irreversible.

First, we wanted to investigate if simulations of the model would mimic known experimental observations. Experiments carried out in cytosolic extracts have shown that upon exogenous application of CytC, maximal caspase 3 (C3*) activation (cleavage of procaspase 3) can occur within 15–30 min in some cells, depending on stimulus strength^11^. Live cell imaging results have also indicated that following initiation, caspase activation is rapidly completed within 16 minutes^49^. Protein concentrations of BAX (0.5 nM), APAF-1 (20 nM), caspase 9 (20 nM), caspase 3 (200 nM) and XIAP (40 nM) previously measured in HeLa cells were assumed^20,43^. A pro-apoptotic insult was stimulated by u = 0.5 nM. In simulations, the initiation of C3* activation occurs after 1000 s (~ 16 min) and takes another 1000 s to complete (Fig. 2A) demonstrating that our model simulations mimic experimental observations from live cell imaging studies^11^.

Apoptosis is generally considered to be irreversible once the executioner caspase 3 is activated beyond a certain threshold^50^, although it must be noted that recent evidence points to the reversibility of apoptosis in some cases^51^. Once caspase 3 is activated apoptosis can happen in a few minutes^52^. For this reason, we wanted to double check the irreversibility of the caspase-activation switch in our model^53^. We carried out a simulation study with differently sized pulse stimulations. Transient pulse stimulations with an amplitude of u = 0.5 nM and durations ranging 300–350 s were used (Fig. 2B). In the model, continuous stimulation of *u* = 0.5 nM activates C3* at 1500 s. Transient pulse stimulation for 310 s (*u* = 0.5 nM) does not result in activation of C3*. But when *u* = 0.5 nM is applied for 315 s, C3* activation switches on to reach ~ 50% of the C3* activation level for continuous stimulation. Increasing lengths of stimulus pulses (320–350 s) increases the C3* activation level. Transient pulse activation of the system results in a lower C3* activation level when compared to the nominal model. However, it is important to note that once the C3* activation threshold of 315 s (~ 5 min) stimulation by *u* = 0.5 nM is reached, C3* activation is irreversible. These results demonstrate that the model that we have developed reflects the experimental observation that apoptosis is irreversible once the activation threshold of caspase 3 is reached. Moreover, they also demonstrate that as shown experimentally^52^, caspase activation is regulated by the duration (Fig. 2B) and strength (Fig. 4A) of the pro-apoptotic signal.

#### The model exhibits bistability

**Figure 3:**
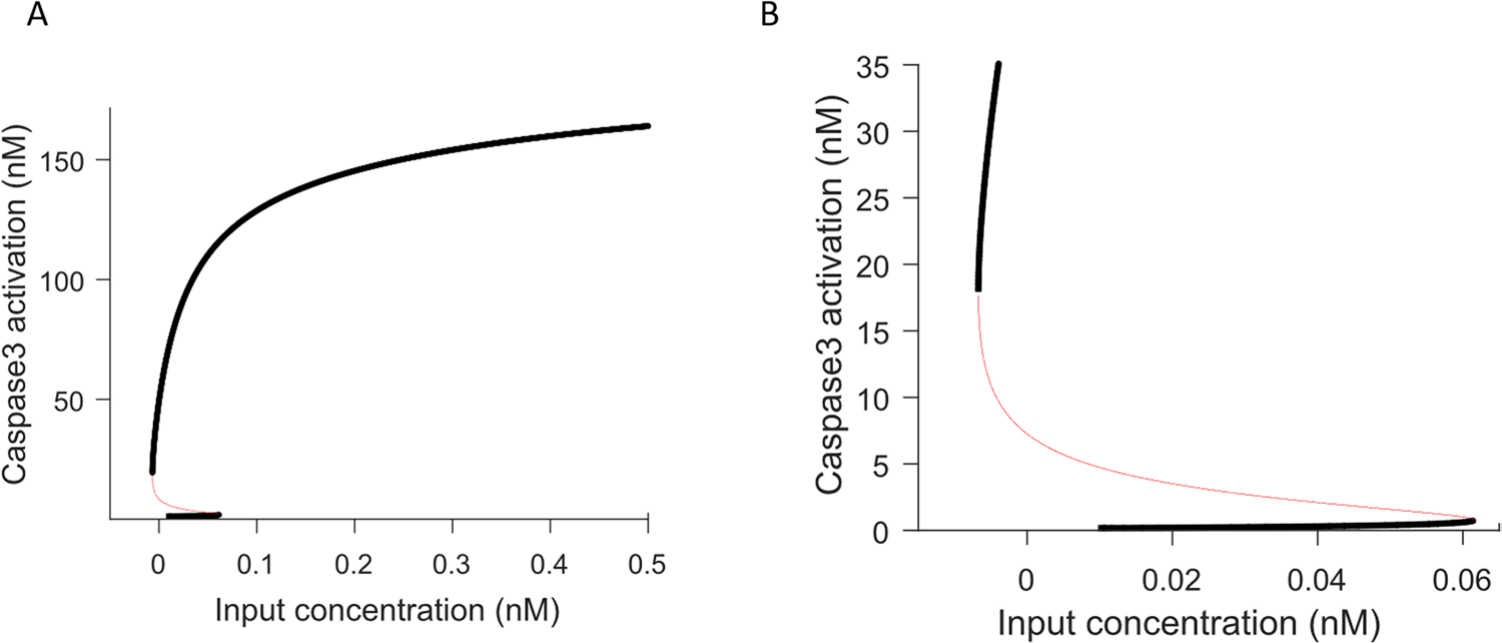
Bistable Behaviour of Caspase 3 activation. Steady state stimulus response bifurcation curve of caspase 3 activation. That simulated caspase 3(C3*) activation demonstrates irreversible bistability and hysteresis. (**A**). Three steady states are exhibited by the system, two stable (solid line) and one unstable (dashed line). The system can reside in one of the two stable steady states (‘on’ or ‘off’) but not in the intermediate unstable steady state. (**B**). The first bifurcation point at 0.06 and the second at − 0.01 indicates that the system displays hysteresis between stimulus concentration (*u*) 0–0.06 nM. The system remains at low caspase 3 activity (‘off’ state) for increasing stimuli until a threshold of *u* (0.06 nM) is reached, at which point caspase 3 activity switches to the second steady state (‘on’ state) irreversibly, in an all-or-none fashion.

The Legewie model showed that XIAP was a mediator of positive feedback causing bistability^20^. This and other models have shown that bistability is a key aspect in the regulation of biological networks and in particular is important for the determination of the irreversibility of apoptosis^19–23^. Therefore, we next wanted to investigate if the addition of BAX and SMAC to our model influenced the bistability and hysteresis previously demonstrated by the Legewie model. To confirm that the model exhibits bistable behaviour we performed a bifurcation analysis. Figure 3A shows two stable (solid line) and one unstable (dashed line) steady states. The system can reside in one of the two stable steady states (‘on’ or ‘off’) but not in the intermediate unstable steady state. In this case, the system remains at low caspase 3 activity (‘off’ state) for increasing stimuli until a threshold of stimulus concentration is reached, at which point caspase 3 activity switches to the second steady state (‘on’ state) irreversibly, in an all-or-none fashion. Critically, in line with time course simulations and literature findings^25,54^, the second bifurcation point of the system is negative (Fig. 3B), which indicates that the activation of C3* is not reversible considering that it is not possible to have negative stimulus concentration. Depending on the state in which the system starts (‘on’ or ‘off’ state), differential response curves will be obtained^20^. Importantly, the first bifurcation point at *u* = 0.06 nM and the second at *u* = -0.01 nM, indicate that the system displays hysteresis between stimulus concentrations of − 0.01 to 0.06 nM (Fig. 3). This is in line with experimental observations which have shown that apoptosis activation depends on the intensity of the stimulus, allowing the cell to survive if the signal is not sufficient to drive the cell to begin programmed cell death^51^.

#### Bax and Smac regulate the timing of the apoptotic switch

**Figure 4:**
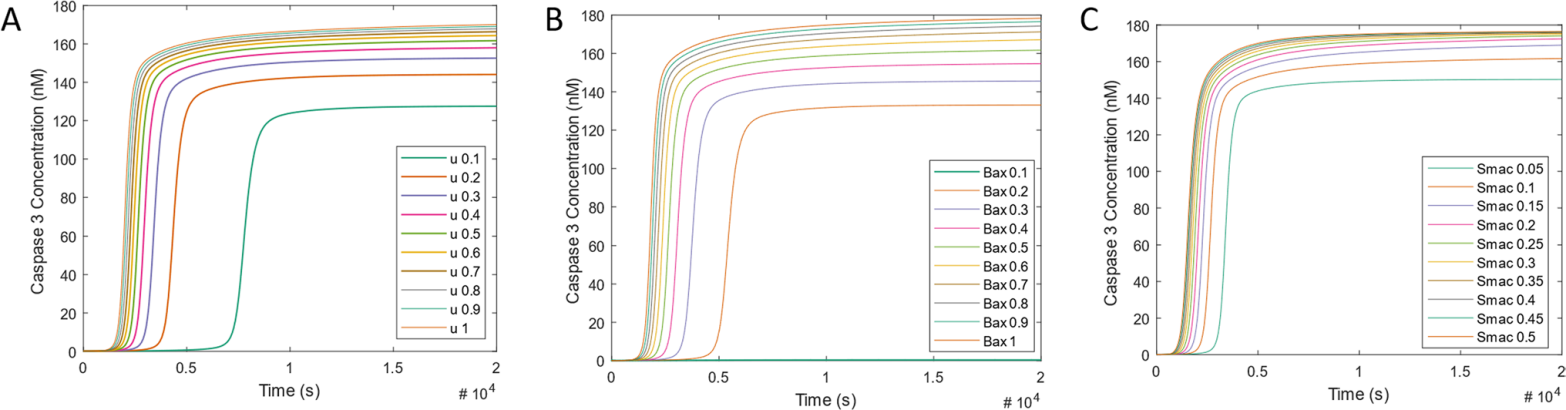
Impact of Input, BAX and SMAC concentration on Caspase 3 activation. Time course activation of caspase 3 upon step-like increase in (**A**). Input stimulus concentration from 0.1–1 nM, (**B**) initial BAX concentration (BAX0) from 0.1–0.9 nM and (**C**) initial SMAC release rate from 0.05–0.5 s^−1^. (**A**) Illustrates caspase 3 activation upon input stimulus variation. Lower *u* increases the caspase 3 activation time-delay while caspase 3 is activated earlier upon increase in *u*. (**B**) indicates that caspase 3 simulated response time and amplitude is inversely related to initial BAX concentration (BAX0). Lowest BAX0 (0.1 nM) results in caspase 3 activation time-delay of 500 s (~ 8 min) with an amplitude of over 130a.u., whereas the highest BAX0 (0.9 nM) results in caspase 3 activation around 100 s (~ 2 min) with an amplitude greater than 160a.u. (**C**) Indicates that caspase 3 activation time-delay and amplitude is similarly inversely related to SMAC release rate. Lowest SMAC release rate (0.05 s^−1^) results in an increase in caspase 3 activation time-delay to a lower amplitude, whereas the highest SMAC release rate (0.5 s^−1^) results in earlier caspase 3 activation with a higher amplitude.

The previous simulation demonstrated that the model that we have built recapitulates the in vivo behaviour of the intrinsic apoptosis pathway. Therefore, we were confident that this model could be used to study the influence of the pro-apoptotic regulators, BAX and SMAC on the mechanistic properties of the activation of the caspase cascade. Hence, we used simulations to investigate how BAX and SMAC control the timing of the apoptotic switch. We first investigated how the strength of the input stimulus (intensity of the signal) controls the observed time-lag of caspase activation (Fig. 4A). A stimulus strength of u = 0.5 nM demonstrated rapid C3* activation in an all-or-nothing fashion. Upon reduction of the input stimulus concentration, C3* does not activate until a later time point, demonstrating an increase in C3* activation time-delay, as is expected for a bistable system. Conversely, increasing the input stimulus concentration reduces the C3* activation time-delay (Fig. 4A). Therefore, we observed that simulation response time is inversely related to the strength of the stimulus.

Next, we investigated whether alteration of BAX and SMAC activity could regulate this time-delay in C3* activity. Altering the initial concentration of BAX (0.1 µM-1 µM) or the release rate of SMAC (0.05 s^−1^–0.5 s^−1^) results in a similar change in C3* time-delay. Higher concentrations of BAX and SMAC result in shorter C3* activation time-delay (Fig. 4B,C). Together, these results show that the time-delay of C3* activation is controlled by (i) the strength of the stimulus, and (ii) the concentration of BAX and SMAC protein expression.

#### Bax and Smac regulate the threshold and amplitude of the apoptotic switch

The results so far indicate that the model that we have generated reflects the behaviour of the intrinsic apoptotic pathway and therefore can be used to analyse the relative contributions of BAX and SMAC to the irreversible activation of the system. Importantly, these proteins have been shown to be deregulated in several diseases such as cancer, where incorrect activation of apoptosis are hallmarks^4,6,27,29,31–40,55–58^. In the case of tumour cells, it has been shown that there is an increase in the threshold of apoptosis activation by downregulation of BAX and SMAC^16,35,39^. Thus, we posit our model could predict how alterations to BAX and SMAC levels can affect the intrinsic apoptosis pathway in cancer cells. To investigate at which precise values transition between stable steady states occur, indicating the threshold of caspase activation, we created bifurcation diagrams for differential concentrations of BAX and SMAC.

**Figure 5:**
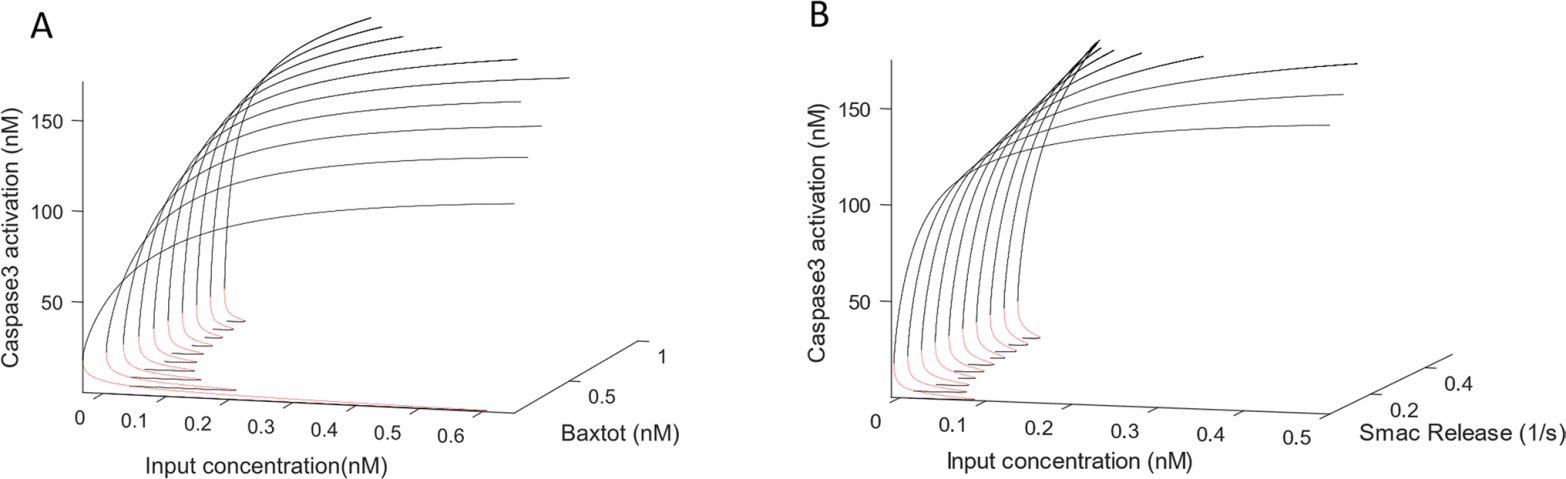
Impact of BAX and SMAC on the Activation Threshold of Caspase 3. (**A**). Bifurcation diagrams for a range of BAX initial concentrations (BAX0) (0.1–1 nM). Nominal BAX0 is 0.5 nM. The first limit bifurcation point of the nominal model with BAX0 at 0.5 nM occurs at *u* (*0*.06 nM)*,* where the system transitions from the ‘off’ state to the ‘on’ state of caspase 3 activity in an all-or-none fashion. Upon decrease of BAX0, a higher limit bifurcation point is observed (*u* (*0*.6 nM) at BAX0 = 0.1 nM). Conversely, with increase in BAX0 to 0.8 nM, a decrease in threshold of caspase 3 activation is observed with a reduced bifurcation point of *u* (0.04 nM). SMAC remained as in the nominal model for all simulations with SMAC release rate = 0.1 s^−1^. (**B**) Bifurcation diagrams for a range of SMAC release rate (0.05–0.5 s^-1^). Nominal SMAC release rate is 0.1 s^−1^. The threshold for activation of C3* in the nominal model, with SMAC release rate (k19) = 0.1 is *u* (0.68 nM). With decrease in SMAC activation by decreasing k19 to 0.01, the threshold for activation of Caspase 3 decreases to *u* (0.9 8 nM) indicating the additional stimulus required to activate C3* upon decreased SMAC levels. With increase in SMAC activation (k19 = 0.1), a downward shift in activation threshold to *u* (0.02 nM) is observed.

First, we investigated how changes in BAX concentration affect the system by performing bifurcation analysis with differential BAX levels. A range of bifurcation plots for differential BAX conditions (BAX0 = 0.1–1 nM) is shown in Fig. 5A. The first limit bifurcation point of the model with BAX0 at 0.5 nM occurs at *u* = 0.068 nM*,* where the system transitions from the ‘off’ state to the ‘on’ state of C3* activity in an all-or-none fashion. With alteration in BAX0, differential system behaviour emerges. Upon decrease of BAX0, in a similar manner to what can be observed in cancer cells such as melanoma and colorectal cancer^6,29,38,40^, a higher limit bifurcation point is observed (u = 0.6 nM at BAX0 = 0.1 nM). This shift in bifurcation point indicates an increase in threshold stimulus (*u*) required for activation of C3* activity upon decrease in BAX0. Conversely, with increase in BAX0 to 0.8 nM, a decrease in threshold of C3* activation is observed with a reduced bifurcation point of u = 0.04 nM. This indicates that with a higher BAX0, there is a reduction in the required stimulus to reach the threshold of activation of C3* and can explain how cell death could be triggered at a lower drug concentration in pathologies where BAX levels are increased. Importantly, this is biologically sound considering that an increase in BAX translocation to the mitochondrial membrane will enhance MOMP, CytC release and reduce the stimulus required to activate the caspase cascade^5^.

Next, we investigated how altered SMAC levels impact the transition between ‘off’ and ‘on’ C3* states. Figure 5B illustrates the C3* bifurcation plots for a range of SMAC values. The parameter ‘k19′ in Table 1 is the activation rate for SMAC and was altered over a range (0.05–0.5 s^−1^) to produce different levels of SMAC. BAX0 remained at 0.5 nM throughout these simulations. The threshold for activation of C3* with k19 = 0.1 s^−1^ is *u* = 0.68 nM). With decrease in SMAC activation by decreasing k19 to 0.05, the threshold for activation of caspase 3 increases to *u* = 0.98 nM indicating the additional stimulus required to activate C3* upon decreased SMAC levels. With increase in SMAC activation, a downward shift in C3* activation threshold to u = 0.02 nM is observed. This implies that with an increase in SMAC levels, by employing a faster activation rate (k19), a lower input stimulus is required to reach the threshold of activation of C3*.

These simulations demonstrate that our model can be used to predict what happens when alterations to SMAC and BAX levels occur in pathological conditions. Importantly, our simulations also showed that while alteration of BAX and SMAC impact the threshold of activation of C3* in a similar pattern, decrease of BAX0 causes a larger increase in the threshold of C3* activation when compared to a decrease in SMAC activation. This would indicate that BAX, but not SMAC, is a more prominent regulator of the apoptotic switch.

Next, we investigated how BAX and SMAC regulate the amplitude of caspase 3 activation. The bifurcation diagrams show BAX affected the C3* values of the ‘on' state more prominently than the SMAC levels. Further, the ‘on’ state of caspase 3 saturates at high input concentrations (u > 0.5 nM). To analyse more quantitatively how alteration of BAX and SMAC impact this saturation amplitude of caspase 3 activation, we simulated dose responses for increasing input concentrations and different BAX and SMAC levels. Firstly, varying the BAX expression levels fivefold down and up (0.1–1 nM) caused variations in the C3* amplitude from 120 to 180 nM (Supplementary Fig. 1A). Note that for lower BAX level (< 0.1) the activation threshold shifts to unphysiologically high input concentrations and C3* does not activate. Secondly, varying the SMAC release rate over a tenfold range (k19, 0.05–0.5 s^−1^) only caused variations in the C3* amplitude from 160 to 180 nM (Supplementary Fig. 1B). Note that decreasing SMAC release rates even lower (< 0.05 s^−1^) C3* does not affect the ability of the system to switch on, nor does it change the C3* amplitudes. Thus, our model predicts that the amplitude of caspase 3 activation is more sensitive to changes in BAX than to changes in SMAC.

Finally, to get a further characterisation of the role of BAX and SMAC in the system we decided to investigate whether alteration of BAX or SMAC impacted the irreversibility of the switch. We studied the second bifurcation point of the system where C3* is transitioning from the high activity ‘on’ state to the ‘off’ state. We observed that alteration of BAX and SMAC does not impact the irreversibility of C3* activation. Although the second bifurcation point increased with increased BAX0 and SMAC, it never became positive indicating that the activation of C3* remains irreversible. Thus, BAX and SMAC do not impact the existence of bistability in our model. In line with other observations in the literature^21^, our results demonstrate that the main function of BAX and SMAC is to control the quantitative bistable properties (such as the “on” state threshold and amplitude), not to generate bistability itself. Bistability of our system emerges as a result of implicit and explicit feedback loops, as in line with the literature^19–21^.

#### Validation experiments confirm that caspase activation is more sensitive to BAX than SMAC

**Figure 6:**
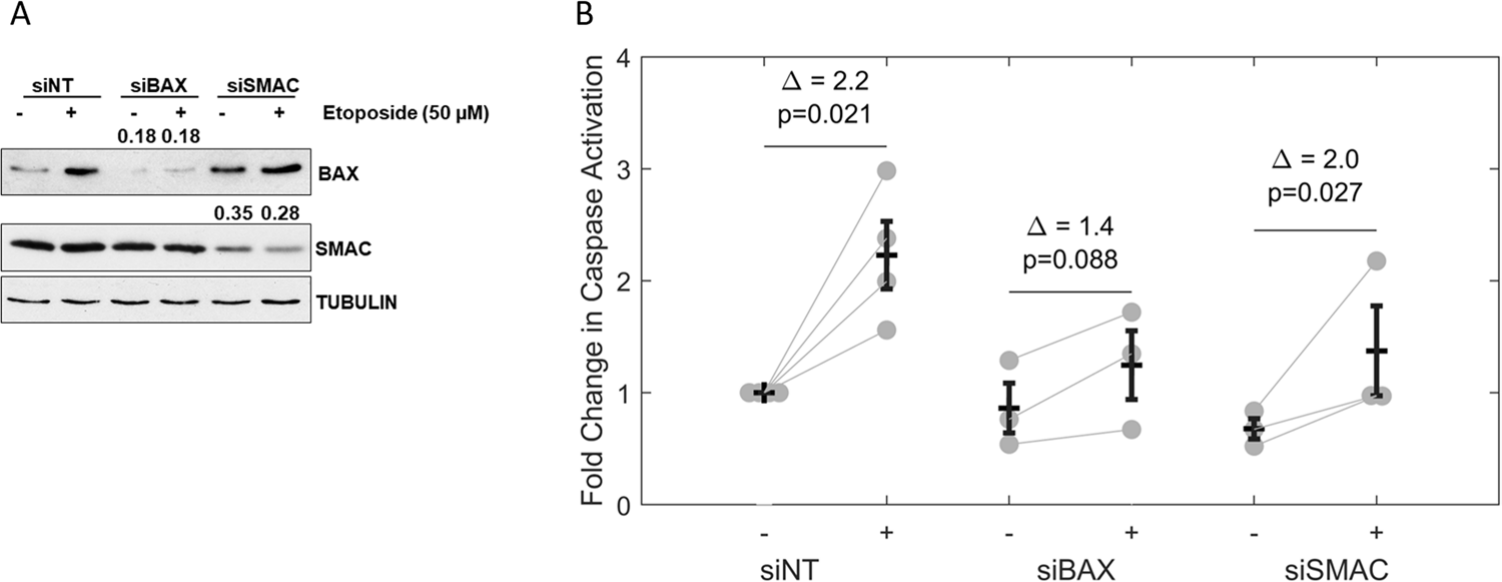
siRNA-mediated downregulation of BAX and SMAC reduces caspase activation upon Etoposide treatment. (**A**) HeLa cells were transfected with either BAX, SMAC or non-target (NT) siRNA and 36 h after transfection the cells were treated with 50 µM Etoposide for 18 h in growing conditions where indicated. Cell lysates were collected, and expression levels of BAX and SMAC proteins were monitored by blotting with specific antibodies. Tubulin α was used as loading control. (**B**) HeLa cells treated as in A were collected from the media or after trypsinization and caspase activation was assessed by FITC-VAD-FMK binding and measured by flow cytometry. The percentage of FITC-VAD-FMK positive cells is represented on the y-axis. Fold change in knockdown is as annotated by Δ. Bars (middle) and error bars (top, bottom) indicate mean + /− standard deviation. In the BAX knockdown, inducing caspase activation with etoposide showed not statistically significant difference compared to DMSO control (p-value = 0.088), whereas in the SMAC knockdown etoposide significantly induced caspase activation (p-value = 0.027) with a fold change similar to the no-knockdown control (1.8 for SMAC knockdown versus 2.0 for siNT control). p-values represent a paired-sample t-test with pairing by experiment (in each experiment the control (−) and knockdown ( +) values were measured).

We performed experiments to validate the most salient model prediction; that alteration of BAX and SMAC expression levels impact the bistable properties of caspase activation. As outlined above, model simulations suggest that the amplitude of caspase activation can be impacted by alteration of BAX, and to a lesser extent SMAC, expression levels. To experimentally validate these model predictions and confirm the role that BAX and SMAC play in controlling the caspase 3 activation, we performed cell culture experiments. In these experiments, reduction of either BAX or SMAC level was achieved by RNA interference (siRNA) and intrinsic apoptosis was induced by the DNA damaging agent Etoposide (50 µM for ~ 18 h). To confirm that the downregulation of BAX and SMAC worked, their protein expression levels were measured by western blot (Fig. 6A). Western blot also demonstrated that upon Etoposide treatment, an increase in BAX protein levels are observed, independently of SMAC levels. This increase in BAX protein levels likely indicate the induction of BAX upon DNA damage to translocate to the mitochondria and initiate the intrinsic caspase cascade.

The corresponding total caspase activity was measured by flow cytometry and is shown in Fig. 6B. Non-transfected control cells show a significant (p-value = 0.021) basal caspase activity fold change of 2 upon application of Etoposide. Upon knockdown of BAX, etoposide induced caspase activation showed no statistically significant difference compared to DMSO control (p-value = 0.088), whereas upon knockdown of SMAC, etoposide treatment significantly induced caspase activation (p-value = 0.027) with a fold change similar to the no-knockdown control (2.0 for SMAC knockdown versus 2.2 for siNT control). These experimental results indicate that the model prediction that BAX has a more pronounced effect on the drug-induced amplitude of caspase activation than SMAC is correct.

#### Simulated caspase activation correlates with drug sensitivity in a panel of melanoma cell lines

To further experimentally validate our model, we next tested whether it could explain the drug-responses of different cell lines. To do this, we analysed publicly available NCI-60 datasets for which proteomic-profiling and drug-response data were available and used the protein expression information to perform cell line specific simulations. These datasets consist of 60 cell lines from 9 tissues, mass-spectrometry based proteome measurements, and measured drug response scores for more than 100,000 drugs, including etoposide^59,60^. BAX but not SMAC levels correlated with etoposide response, Pearson correlation coefficient (C) = 0.307, p-value (p) = 0.019, (Supplementary Fig. 3), suggesting that BAX but not SMAC protein levels provide useful information to personalize the model to the individual cell lines. Similarly, the concentration of SMAC in our model had little effect the amplitude of caspase activation compared to BAX, and the size of this effect remained small for different XIAP concentrations (Supplementary Fig. 5). Thus, we focused on BAX to generate cell-line specific simulations. The measured SWATH values for BAX were used to adjust the BAX total protein expression parameter in the model as follows; if for example BAX levels were 1.5 times increased in cell line A, then we increased the corresponding BAX protein expression parameter (k1) by a factor of 1.5. The resulting set of cell-line specific parameters was then used to simulate the caspase activation trajectories for each cell line.

**Figure 7:**
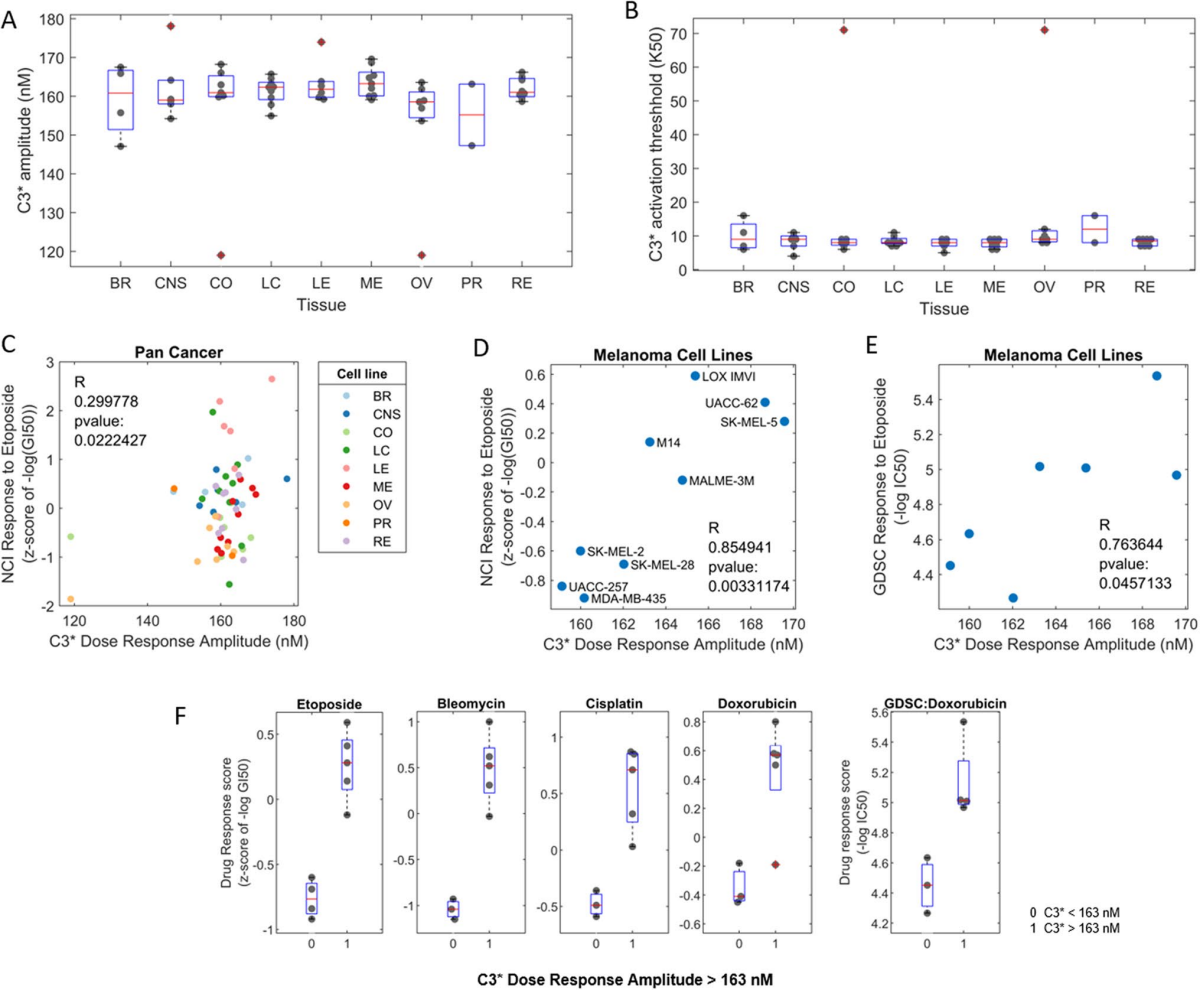
Cell Line Specific Simulations Correlate with Drug Response. (**A**,**B**) Distributions of the cell-line specific simulated caspase 3 activation amplitudes (**A**) and caspase 3 activation thresholds (**B**) for the issues indicated on the x-axis. Boxplots indicate the upper and lower quartile (blue box) and median (red line). Dots represent the simulated values for each cell line. *BR* breast, *CNS* central nervous system, *CO* colorectal, *LC* lung cancer, *LE* leukaemia; *ME* melanoma, *OV* ovarian cancer, *PR* prostate, *RE* renal cancer. (**C**) Pan cancer cell line specific simulations were carried out and C3 amplitude was correlated with cell line response to Etoposide (C = 0.3, p-value = 0.022). (**D**) Melanoma cell line specific simulations (n = 9) indicated a positive correlation with response to etoposide measured by the NCI-60 screen (C = 0.855, p-value = 0.003). (**E**) Melanoma cell line specific simulations (n = 7) are positively correlated with etoposide response measured in the GDSC screen (C = 0.76, p-value = 0.046). (**F**) Classification of the melanoma cells into sensitive and insensitive cell lines. Shown are the distributions of the indicated drug responses (y-axis) for cells with a low simulated caspase 3 activation amplitude (< 163 nM) on the left (0) and cells with a high amplitude (> 163 nM) on the right (1). Boxplots indicate the upper and lower quartile (blue box) and median (red line). Dots represent the values for each cell line.

The simulation results showed that the C3 amplitude and activation-threshold varied markedly between cell-lines (ranging from 120 to almost 180 nM, Fig. 7A,B) and correlated with the etoposide response (C = 0.3, p-value = 0.022, Fig. 7C). This correlation between the C3 amplitude and the etoposide response might be a tissue effect, meaning that one tissue is sensitive to etoposide, but another is not. To check this, we looked at the variability of the simulated responses across tissues but observed no pronounced differences of the C3 amplitude between different tissues (Fig. 7A). This indicates that the observed correlation between the C3 amplitude and the etoposide response depends on the specific genomics of each cell-line and not tissue-specific differences.

Another explanation for the correlation might be that the model explains the etoposide response very well in one tissue but not the others. To test this, we analysed the simulations for each tissue separately, focusing on those tissues with more than 5 cell lines (Table 3). The best correlation between the simulated C3 amplitude and the etoposide response was observed in melanoma cell lines, C = 0.855, p-value = 0.003, n = 9 cell-lines (Fig. 7D). To confirm this, we also analysed data from an independent drug screen (GDSC^61^), and found a similar correlation C = 0.76, p-value = 0.046, n = 7 (Fig. 7E). Ovarian cancer cell lines also exhibited a correlation, C = 0.77, p-value = 0.04, n = 7 (Figure S3). The correlations for the other tissues (leukemia, colorectal, CNS, breast, renal, lung and prostate) were weak and not significant (Table 3).

**Table 3:** Table of cell lines used for cell line specific simulations.

Tissue	Correlation (C)	P-Value
Leukemia	0.575	0.233
Colorectal	0.046	0.923
CNS	0.428	0.397
Breast	0.352	0.648
Renal	− 0.237	0.572
Lung	− 0.362	0.339
Ovarian	0.759	0.048
Melanoma	0.845	0.004

Focusing on the melanoma cells, we wanted to test how general the model is with respect to other DNA damaging drugs and analysed the data for bleomycin, cisplatin, and doxorubicin. For all three drugs, the drug response scores correlated with the simulated C3 amplitude (Figure S3). The correlation of the C3 amplitude with the bleomycin and doxorubicin responses was particularly strong and significant (C = 0.77, p-value = 0.03 and C = 0.79, p-value = 0.02, respectively). The results suggest that the model can be used to simulate and predict caspase activation and the responses to DNA damaging drugs in melanoma cells.

Given the observed correlation between the simulated C3 amplitude and the response to DNA damaging drugs, we asked whether a particular threshold in the model could distinguish between sensitive and resistant cells. We found that for all analysed drugs, a threshold value for the C3 amplitude of 163 nM was able to distinguish between sensitive and resistant cells (Fig. 7F). All melanoma cells with a simulated C3 amplitude less than 163 nM were sensitive to etoposide, bleomycin, cisplatin and doxorubicin, with NCI-60 response scores < 0 (n = 8 cells). Conversely, the cell lines with a simulated C3 amplitude greater that 163 nM were resistant to all three drugs. Only one outlier for doxorubicin was observed, where M14, was falsely classified as sensitive (Fig. 7F). In summary, our results suggest that BAX protein levels can be used to personalize the model, simulate caspase activation, and predict drug-responses for DNA-damaging drugs in melanoma cells. A defined threshold value of 163 nM of caspase activation in the model distinguishes between sensitive and resistant melanoma cells.

## Discussion

In the current work we have developed a mathematical model of the intrinsic apoptotic pathway that recapitulates the behaviour of this systems in cells. The current model includes BAX and SMAC, two nodes that have not been widely analysed in previous computational models but are *bona fide* regulators of this network. Importantly, the addition of these nodes to the model and their experimental validation is a clear advance of our study with respect to other models, and better recapitulates the behaviour of the system in cells enhancing the predictive power of the computational model. The most salient model prediction was that BAX exerts stronger control over the amplitude of the apoptotic switch than SMAC, and our cell culture experiments validated this prediction. In our validation experiments, etoposide induced a significant fold-change of the measured caspase activation in both the control and the SMAC knockdown, but not in the BAX knockdown, thus validating the model prediction. Interestingly, the BAX and SMAC knockdowns also exhibited a trend in decreasing the basal, unstimulated caspase activation (Fig. 6B) in our experiments. This basal, unstimulated caspase activity is not described by the current model, which focusses on drug-induced caspase activation, and might provide an opportunity for future model extensions.

Using measured BAX protein levels to personalise the model to each cell line of the NCI-60 panel, it was revealed that the simulated caspase activity correlated with the cell line response to etoposide. Further, a distinct threshold of caspase 3 activation separated sensitive from insensitive melanoma lines for a variety of DNA damaging drugs. This finding is particularly interesting in cancer and provides a possible explanation for why the deregulation of BAX is more common than deregulation of SMAC in cancer^5,62,63^.

Importantly, our model confirms the existence of bistability in accordance with other studies of the apoptosis network^20,21^. However, many studies modelling apoptosis focused on the extrinsic pathway consisting of death receptor activation, subsequent activation of caspases and secondary activation of the mitochondrial pathway^22,23,41,64^, or the processes upstream of caspase activation such as BCL2 protein regulation of MOMP^21–23,65,66^. Other modelling studies that concerned the activation of caspases in the intrinsic pathway did either not look at the control of bistable properties^22,64,65^, or did not include BAX and SMAC^19,20,23^. A notable exception is the work by Tyson and colleagues^21^, which provided and excellent account of which network nodes and parameters regulate the bistability properties, in particular irreversibility, time-delay and threshold. They found that the time-delay and threshold are primarily controlled by the initiator module (corresponding to BAX in our model), which is in accordance with our findings. However, we also find that SMAC, which merely relayed the signal in the Tyson model, markedly controlled the delay and threshold of caspase activation. Further, and in contrast to Tyson, we also analysed how BAX and SMAC control the amplitude of the apoptotic switch, and experimentally validated this prediction.

The results from our simulations clearly indicate that the modification of BAX and SMAC levels have a direct effect on the threshold and time delay of caspase 3 activation and determine when the system reaches the point of no return. Thus, the simulations show that decrease of both proteins results in an increase of this threshold of apoptosis, i.e. lowering the expression of these proteins makes the system more resistant to pro-apoptotic stimuli. The fact that the personalised simulations of caspase 3 activity correlated with the response to DNA damaging drugs in the NCI-60 pan cancer dataset suggests that the model can be used to predict the individual cellular responses of different cell lines and patients. This could work particularly well for melanoma, where the cell-line specific simulations confirmed the existence of a distinct threshold separating sensitive from resistant melanoma cells. To improve the model for other tissues, further key nodes that regulate the caspase system in addition to BAX could be tested, mathematically modelled, and connected to the current model. Prime candidates are members of the BCL2 family and a more detailed model of the outer mitochondria membrane permeabilization that have proven predictive power in triple negative breast and colorectal cancer^42,67,68^. Personalising this model with experimental and clinical data could be used to carry out in silico drug targeting and could help the development of novel therapeutics for cancers where the model is predictive, in particular melanoma. Importantly, our simulations indicate that critical bistable properties such as the amplitude of the “on-state” are more dependent on variations of the level of BAX than SMAC. Taking into account that there are currently different SMAC and BAX mimetics for the treatment of cancer at different stages of development, our model would support the idea that BAX mimetics would be more effective than SMAC mimetics.

In summary we present a new experimentally validated mathematical model that can be used to study the dynamics of activation of apoptosis by the mitochondrial pathway.

## Materials and methods

### Model construction and simulation

Our core model is based on a well-established model of caspase bistability in the literature^20^ and has been extended to include BAX and SMAC and the associated reactions (see main text, Fig. 1). Based on this reaction kinetic scheme an ODE model has been constructed using the principle of mass balance and the appropriate reaction kinetic laws, as is common practice^69^. All ODE simulations were carried out using the MATLAB environment (R2018a). Bifurcation analyses were carried out using XPPAUT (http://www.math.pitt.edu/~bard/bardware/xpp/xpp.html) and plotted using the plotxppaut plugin for MATLAB(http://www2.gsu.edu/~matrhc/XPP-Matlab.html).

### Cell culture

HeLa cells (ATTC) were grown in DMEM (Gibco) supplemented with 10% FBS (Gibco) and 2 mM glutamine (Gibco) at 37 °C, 21% O_2_ and 5% CO_2_ in a humidified incubator and kept in exponentially growing conditions. Lipofectamine 2000 (Invitrogen) was used for siRNA transfection according to manufacturer’s protocol. Briefly, cells were seeded 24 h prior to transfection (70% confluency) and 6.25 µL of Lipofectamine 2000 and 100 pmol of siRNA per 60 mm petri dish were used. 24 h after transfection, cells were treated with 50 µM of Etoposide (Sigma Aldrich) or vehicle (DMSO) for 18 h. siRNAs against human BAX and SMAC and a non-target siRNA control were purchased from Dharmacon (ON-TARGETplus Human BAX (581) siRNA–SMARTpool, ON-TARGETplus Human DIABLO (56,616) siRNA–SMARTpool and ON-TARGETplus Non-targeting siRNA #1, respectively).

### Caspase activation assay

Caspase activation was measured in an Accuri's C6 Flow Cytometer System using the CaspACE-FITC-VAD-FMK kit (Promega). Alive and dead cells were collected, centrifuged for 5 min at 300×*g* and incubated with FITC-VAV-FMK in serum free media (1:2000) for 30 min at 37 °C protected from light. Cells were washed once with PBS and incubated with PI for 5 min at room temperature prior to acquisition. FITC and PI emissions were measured using the 533/30 and 670LP detectors respectively following excitation by 488 nm laser. Statistical analysis was carried out to determine the mean caspase activation and standard deviation within each group (siNT, siBAX and siSMAC), control and treated. Fold change in caspase activation was calculated for each treated group compared to the group control.

### Western blot

Cells were lysed in a buffer containing: 20 mM Hepes pH 7.5, 150 mM NaCl, 1% NP40 supplemented with protease and phosphatase inhibitors (Roche). Protein extracts were clarified by centrifugation (14,000 rpm, 4 °C, 10 min) and pellet discarded. Protein quantification was carried out using the BCA protein Assay kit (23225-Invitrogen) according to manufacturer’s protocol. Primary antibodies (1:1000 dilution): anti-SMAC Y12 (ab32023-Abcam), anti-BAX 6A7 (556467-BD Pharmingen), Tubulin TU-U2 (sc-8035-Santa Cruz).

### Cell line specific simulations

BAX and SMAC protein expression (SWATH values^70,71^), GDSC Etoposide response and NCI-60 drug response to Etoposide, Doxorubicin, Bleomycin and Cisplatin were downloaded using the CellMiner tool (https://discover.nci.nih.gov/cellminercdb/, (29/11/2019). All information was collated and stored in a single file. The measured SWATH values of the BAX protein levels swaBAX (log10) were mean normalised $relBA{X}_{i}= swaBA{X}_{i}-mean(swaBA{X}_{i})$ used to adjust the $BA{X}_{tot}$ parameter for each cell line $i$ as follows $BA{X}_{tot,i} = {BA{X}_{tot}\cdot 10}^{relBA{X}_{i}}$, with the nominal BAX protein expression parameter of $BA{X}_{tot}=0.5.$

## Supplementary Information

Supplementary Information

## Data Availability

The MATLAB and XPPAUT files and relevant NCI-60 data of the model are available in the Supplement. SBML version of the code is available at EMBL-EBI BioModels (accession MODEL2001130002)^72^.
