# Supplementary material for: BAX and SMAC regulate bistable properties of the apoptotic caspase system
Source: Sci Rep. 2021 Feb 8;11:3272. doi: 10.1038/s41598-021-82215-2 (PMC7870884; doi:10.1038/s41598-021-82215-2)
Supplement: Supplementary file 1 — Supplementary Information [file 41598_2021_82215_MOESM1_ESM.docx]

BAX and SMAC Regulate Bistable Properties of the Apoptotic Caspase System

Stephanie McKenna^1^, Lucía García-Gutiérrez^1^, David Matallanas^1,2^*, Dirk Fey^1,2^* (*corresponding and senior authors)

1 Systems Biology Ireland, University College Dublin, Belfield, Dublin 4, Ireland

2 School of Medicine, University College Dublin, Belfield, Dublin 4, Ireland

e-mail:

David Matallanas: [david.gomez@ucd.ie](mailto:david.gomez@ucd.ie)

Dirk Fey: [dirk.fey@ucd.ie](mailto:dirk.fey@ucd.ie)

# Supplementary Information

Supplementary Figure 1.
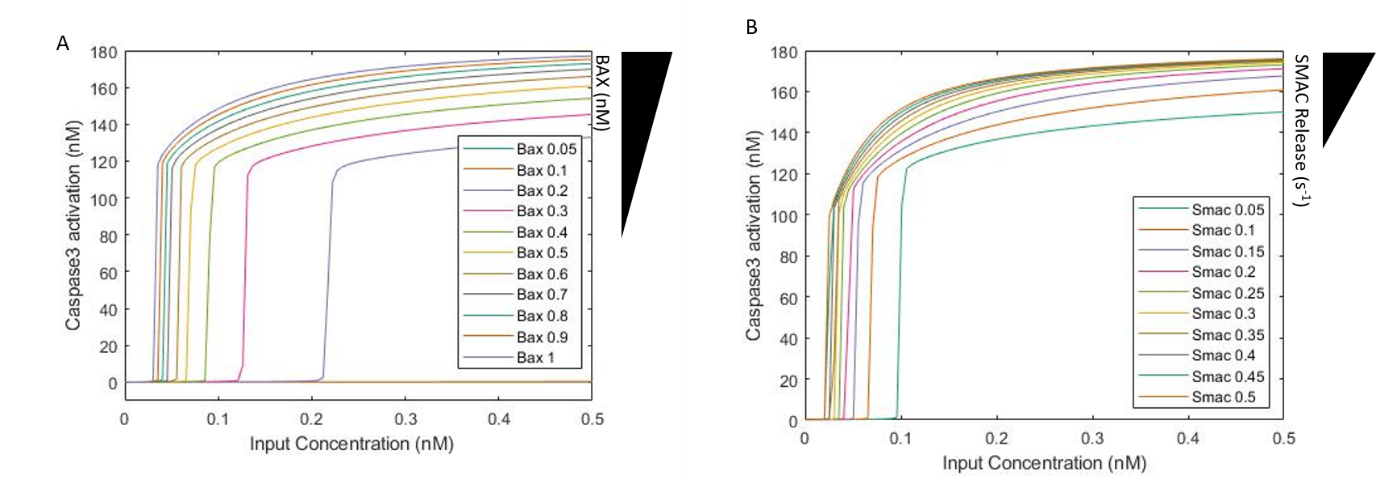


(A) Dose response simulations of caspase 3 activation for a range of BAX initial concentrations (BAX0) (0.1-1nM). Nominal BAX0 is 0.5nM. The concentration of input stimulus required to activate caspase 3 decreased with increase in BAX0. SMAC remained as in the nominal model for all simulations with SMAC release rate =0.1s^-1^.
(B) Dose response simulations for a range of SMAC release rates (0.05-0.5s-1). Nominal SMAC release rate is 0.1 s^-1^. Upon increase in SMAC release rate, a decrease in concentration of input stimulus required to activate caspase 3 is observed.

Supplementary Figure 2. Sensitivity Analysis of Model Parameters.


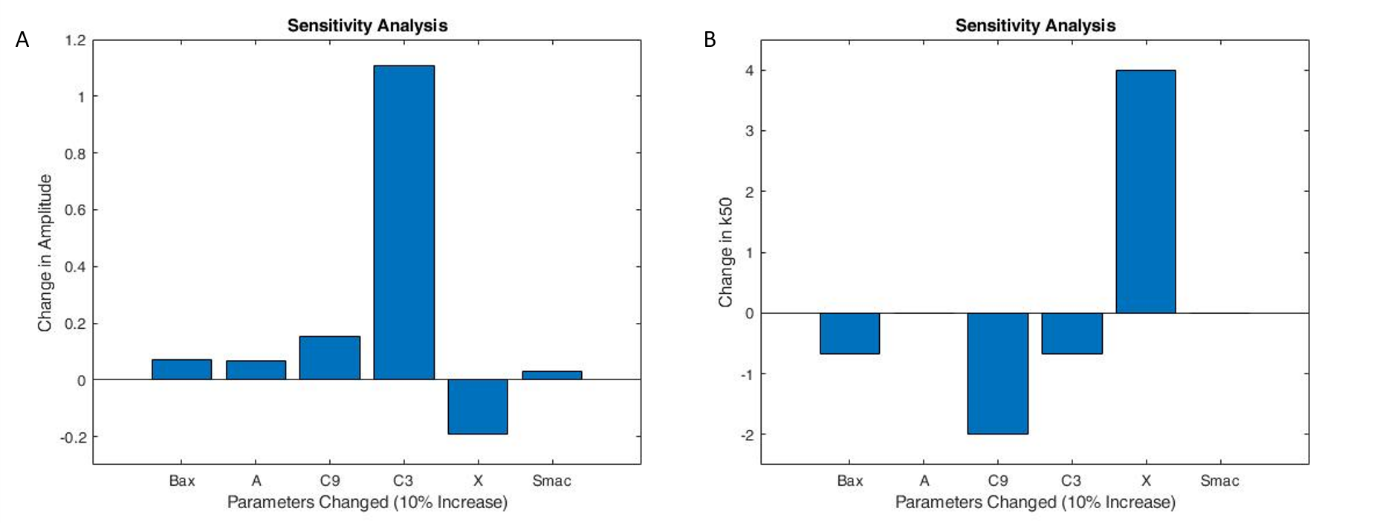


A sensitivity analysis was carried out for the model species. Parameters were increased by 10%, model simulations were run and results were normalised by the change in parameter. (A) It emerged that a 10% increase in Caspase 3 concentration had the highest impact on C3 amplitude. (B) A 10% increase in XIAP concentration had the highest impact on C3 activation threshold.

Supplementary Figure 3. Cell Line Specific Simulations and Correlation with Drug response.


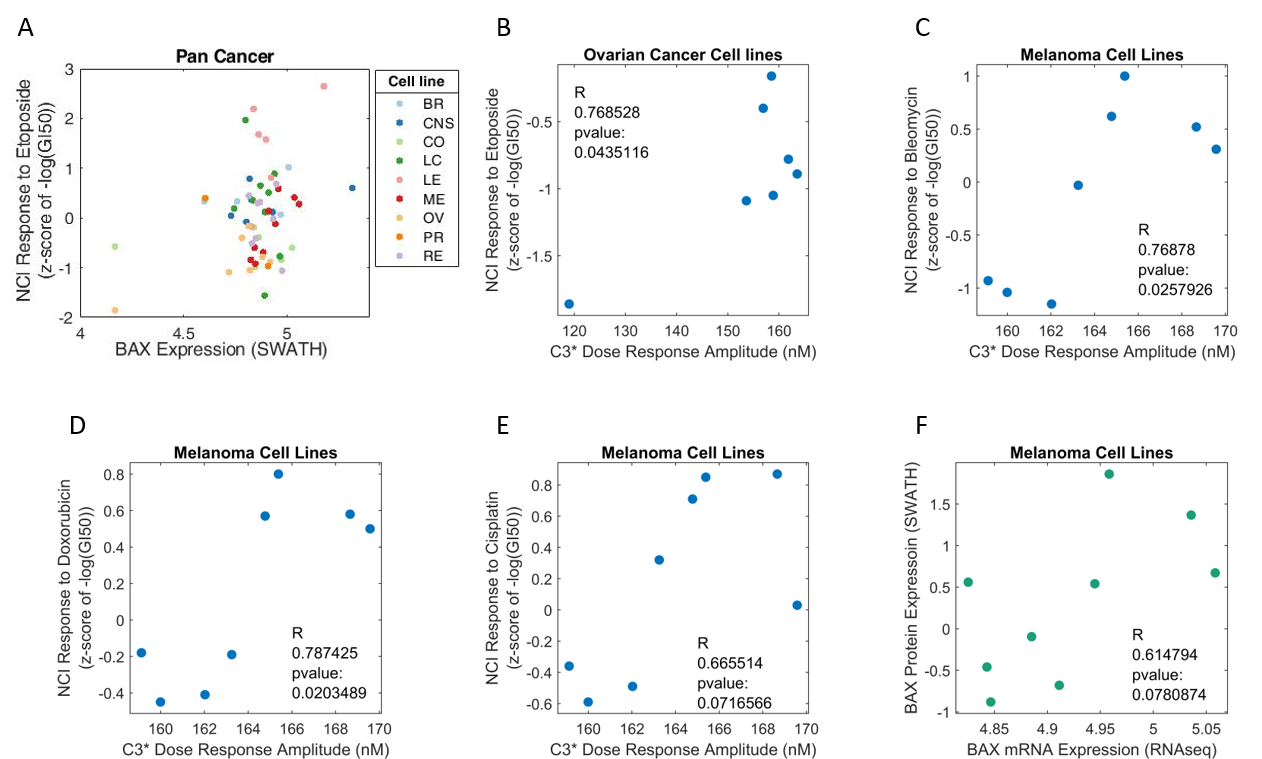


A. Pan cancer BAX levels (protein SWATH) positively correlate with response to Etoposide (C=0.307, pval=0.019). B. Ovarian cancer cell line specific simulation C3 activation amplitude positively correlates with ovarian cell line response to Etoposide (C=0.77, pval=0.04). C-E. Melanoma cell line specific simulation C3 amplitude demonstrates positive correlations with alternative DNA damaging agents: Bleomycin, Doxorubicin, Cisplatin. F. BAX mRNA expression and protein (SWATH) levels are positively correlated (C=0.615, pval=0.078).

Supplementary Figure 4. Original Western Blot


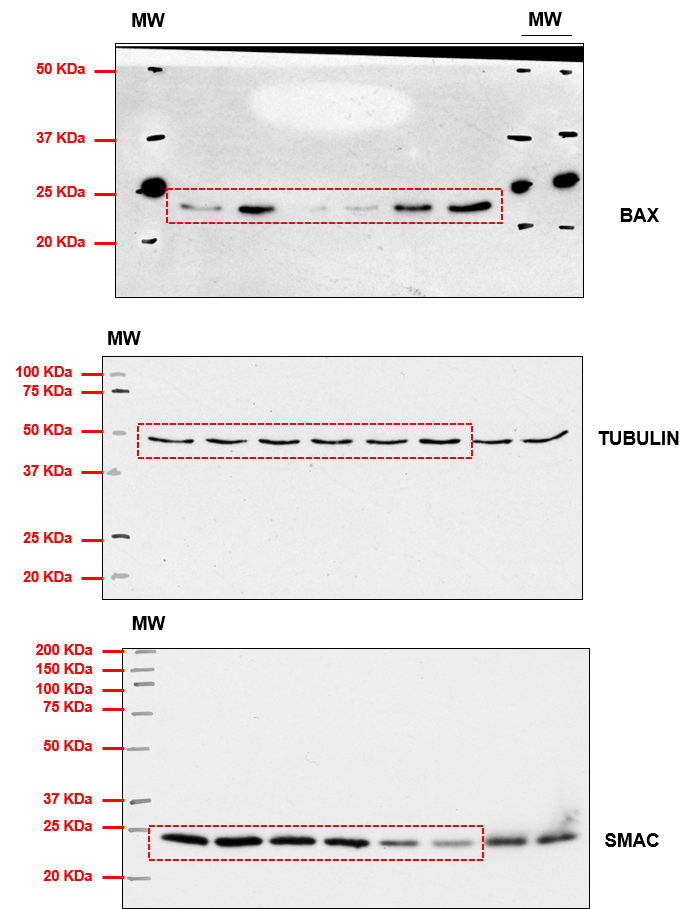


Blots corresponding to figure 6a. BAX and SMAC were run in different gels and only one blot for tubulin is shown. Levels of tubulin from the gel corresponding to BAX are shown below. MW: Molecular weight marker.

Supplementary Figure 5. Impact of Reduced XIAP on Caspase 3 Activation


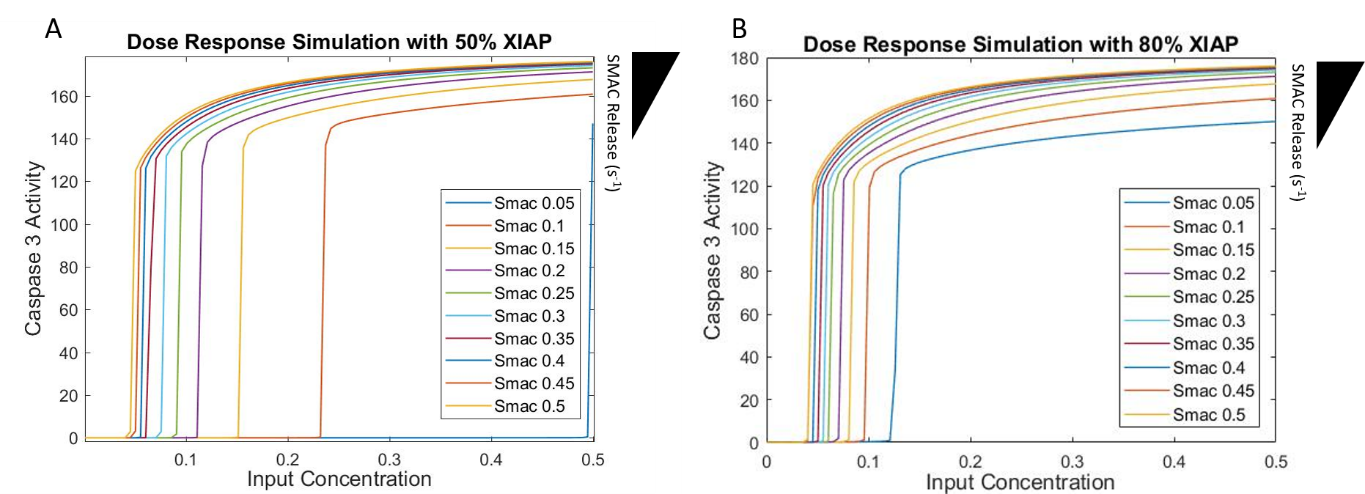


Dose response simulations for various concentrations of SMAC were carried in the presence of (A) 50% XIAP or (B) 80% XIAP. It emerged that upon 50% decrease in XIAP, SMAC had more of an impact on the C3* activation switch.

Supplementary Figure 6. Relationship Between BAK Expression and Response the Etoposide.


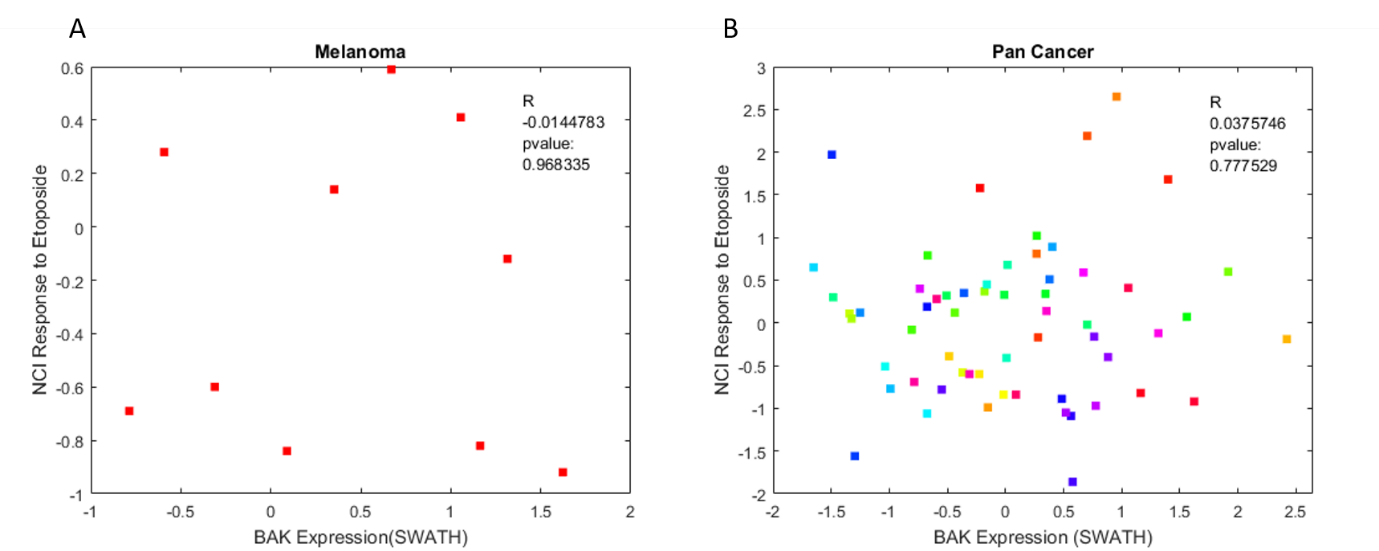


The correlation between BAK expression and response to Etoposide in the (A) Pan Cancer and (B) Melanoma NCI-60 cell line panel was investigated. No significant correlation was observed.

Supplementary Figure 7. Relationship Between BCL2 Expression and Response the Etoposide.


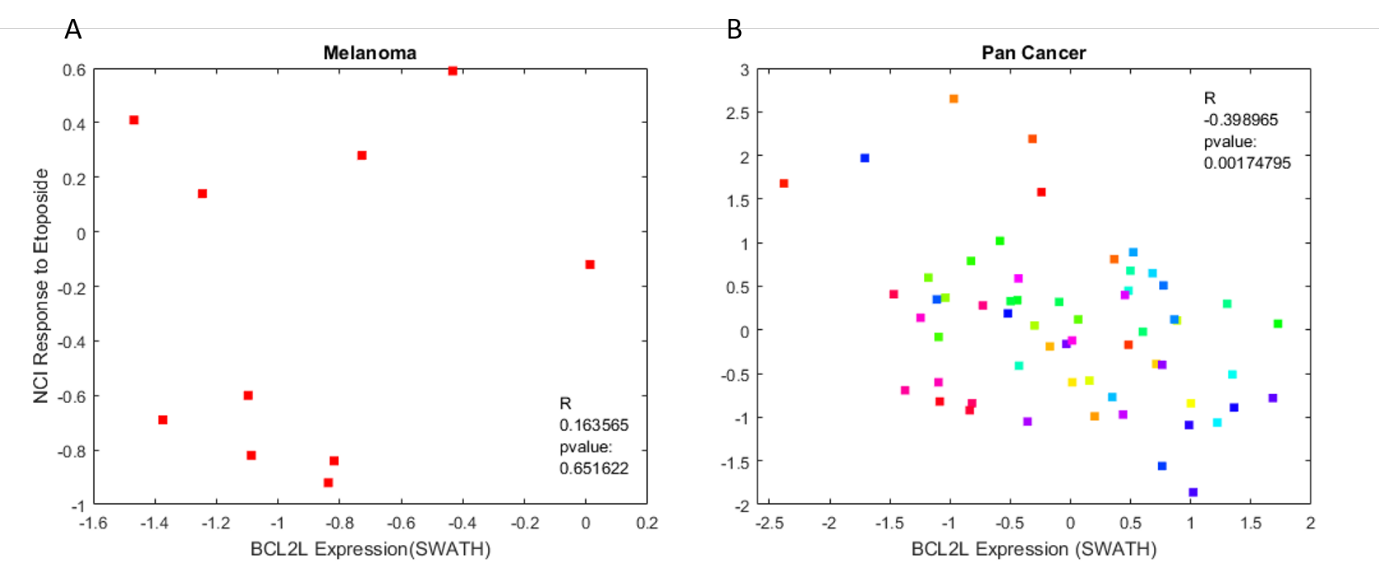


The correlation between BCL2 expression and response to Etoposide in the (A) Pan Cancer and (B) Melanoma NCI-60 cell line panel was plotted. No significant correlation between the anti-apoptotic protein and response to Etoposide was observed.
